# Glomerular and Tubular Renal Function after Repeated Once-Daily Tobramycin Courses in Cystic Fibrosis Patients

**DOI:** 10.1155/2017/2602653

**Published:** 2017-01-04

**Authors:** Florian Stehling, Rainer Büscher, Jörg Grosse-Onnebrink, Peter F. Hoyer, Uwe Mellies

**Affiliations:** ^1^Pediatric Pulmonology and Sleep Medicine, University Children's Hospital Essen, Hufelandstr. 55, 45147 Essen, Germany; ^2^Pediatric Nephrology, University Children's Hospital Essen, Hufelandstr. 55, 45147 Essen, Germany; ^3^Department of General Pediatrics, Pediatric Respiratory Medicine Unit, University Children's Hospital Muenster, Muenster, Germany

## Abstract

*Introduction*. Antibiotic treatment regimens against* Pseudomonas aeruginosa* lung infection in cystic fibrosis (CF) patients often include aminoglycoside antibiotics that may cause chronic renal failure after repeated courses. Aminoaciduria is an early marker of acute aminoglycoside-induced renal tubular dysfunction. We hypothesized that urinary amino acid reabsorption is decreased after repeated once-daily tobramycin therapies.* Methods*. In this prospective cross-sectional study creatinine clearance was estimated by the Schwartz and the Cockcroft-Gault formula. Tubular amino acid reabsorption was determined by ion exchange chromatography in 46 patients with CF who received multiple tobramycin courses (6.3 ± 10.1 (1–57)) in a once-daily dosing regimen and 10 who did not.* Results*. Estimated creatinine clearance employing the Cockcroft-Gault was mildly reduced in 17/46 (37%) of the patients who received tobramycin and 5/10 (50%) of the patients who did not but in none using the Schwartz formula. No association with lifetime tobramycin courses was found. Tubular amino acid reabsorption was not influenced by the amount of once-daily tobramycin courses.* Conclusion*. Clinically not significant reduction of eCCL occurred in a minority of CF patients. However, chronic tubular dysfunction was not present in patients with CF repeatedly treated with tobramycin in the once-daily dosing scheme.

## 1. Introduction

Cystic fibrosis (CF) is the most common autosomal recessive inherited disease in the Caucasian population, and morbidity and mortality in these patients are mainly determined by the course of CF lung disease. Chronic lung infection with* Pseudomonas aeruginosa* is known to worsen prognosis substantially [1]. The only therapeutic option for end-stage lung disease in patients with CF is lung transplantation [2]. Extensive antibiotic regimens to control* Pseudomonas aeruginosa* infections usually include aminoglycosides that are known to cause acute and chronic renal failure.

Attention for CF associated morbidities as chronic renal dysfunction is increasing [3]. The CF transmembrane regulator (CFTR) protein is expressed in the proximal and distal kidney tubules [4], but its impact on renal function remains to be elucidated. Results from autopsies on patients with CF [5] and renal biopsies [6] have revealed different renal pathologies, including proliferation of lysosomal bodies which has been attributed to aminoglycoside nephrotoxicity [5]. Repeated courses of aminoglycoside antibiotics may cause chronic renal insufficiency [7], but data are controversial [8]. Those results are derived from retrospective data during the era when aminoglycosides were given three times daily. Since the early 2000s most CF centres adapted to the once-daily aminoglycoside dosing regimen with the aim of reducing nephrotoxicity [9].

Aminoglycoside-induced acute renal injury may be associated with partial or global renal tubular dysfunction, that is, Fanconi syndrome leading to tubular loss of magnesium that may cause systemic hypomagnesemia [10], glucose excretion, bicarbonate excretion, or phosphate loss. However, these biochemical features may be influenced by CF-related comorbidities, including CF-related diabetes mellitus, hypoventilation, or osteoporosis. On the contrary, amino acid tubular reabsorption is not affected by CF-related comorbidities but may also emerge under aminoglycoside nephrotoxicity. Hyperaminoaciduria has been shown to be an early and sensitive marker of acute aminoglycoside-induced renal tubular damage in animal models and humans [11, 12], which precedes elevation of conventional renal injury markers [13]. Therefore hyperaminoaciduria may be an ideal marker for aminoglycoside-induced kidney injury.

The aim of our prospective cross-sectional study was to analyse glomerular and tubular renal function in patients with CF treated with repeated courses of once-daily tobramycin evaluating estimated GFR and tubular amino acid reabsorption. We hypothesized that repeated courses of once-daily tobramycin result in hyperaminoaciduria.

## 2. Methods

### 2.1. Study Population

Our cross-sectional study population consisted of 56 subjects with CF treated in outpatient clinic of the University Children's Hospital in Essen, Germany, between February 2006 and July 2008. The urine and plasma samples were taken simultaneously during the annual blood collection and immediately analysed. All subjects were in a stable clinical condition and did not receive intravenous tobramycin for four weeks preceding this study. No subject had a history of kidney injury or acute drug-related nephrotoxicity. Renal ultrasound scans were normal in all subjects at annual screenings preceding this study.

### 2.2. Assessment of Renal Function

Estimated creatinine clearance (eCCL) was calculated according to the formula of Schwartz and Cockcroft-Gault. Using the Schwartz formula usually employed to calculate eCCL in children serum creatinine is adjusted for height by the formula* k ∗* length (cm)/plasma creatinine, with* k* = 0.55 for girls and boys up to the age of 12 and* k* = 0.70 for adolescent boys 13 years or older [14]. The second formula of Cockcroft-Gault used to calculate eCCL in adults refers to the following: eCCL = (140 − age) *∗* weight [kg]/72 *∗* SCr [mg/dL] *∗* (0.85 for females) [15]. Renal function was classified according to the KDIGO (kidney disease improving global outcome) guidelines: G1 (normal or high) = GFR > 90 mL/min/1.73 m^2^, G2 (mildly decreased) = GFR 60–89 mL/min/1.73 m^2^, G3a (mildly to moderately decreased) = GFR 45–59 mL/min/1.73 m^2^, G3b (moderately to severely decreased) = GFR 30–44 mL/min/1.73 m^2^, G4 (severely decreased) = GFR 15–29 mL/min/1.73 m^2^, and G5 (kidney failure) = GFR < 15 mL/min/1.73 m^2^ [16]. Both formulas were applied to the entire cohort, since the ages spanned the transition zone between childhood and adulthood.

### 2.3. Assessment of Renal Tubular Function

For the assessment of renal tubular function, plasma and urine samples were collected at the same day, deproteinized within 30 min. and immediately analysed without storage. Free amino acid concentrations (threonine, serine, glycine, alanine, valine, cysteine, isoleucine, leucine, tyrosine, phenylalanine, ornithine, lysine, histidine, and arginine) were determined by ion exchange chromatography using an LC 3000 amino acid analyzer (Eppendorf, Hamburg, Germany) according to manufacturer's specifications. Results are expressed as *μ*mol/l. The excreted amino acids in the urine were normalized to urine creatinine concentration. Tubular reabsorption (in %) was calculated according to the formula 1 − (urine amino acid (*μ*mol/L) × serum creatinine (*μ*mol/L))/(plasma amino acid (*μ*mol/L) × urine creatinine (*μ*mol/L)) × 100.

The ethics committee of the University of Duisburg-Essen approved this study, and parents and/or subjects provided informed written consent before their involvement.

### 2.4. Statistics

Statistical analysis was performed using version 22 of the SPSS Statistics package (SPSS Inc., Chicago, USA). Statistical significance testing was done exploratively without analysis for multiple testing. Intergroup analysis was done employing the Mann–Whitney *U* test, as some of the parameters were not normally distributed. Linear regression analysis with the number of intravenous aminoglycoside courses defined as the dependent variable was employed to show an association between the cumulative aminoglycoside exposure and renal function. To exclude an age dependency we continued with univariate analysis with the intravenous aminoglycoside courses as dependent variable, tubular reabsorption as independent variable, and age as cofactor.

## 3. Results

In our study subjects treated with aminoglycosides (=Tob+ group) were older and had advanced CF disease with chronic* Pseudomonas aeruginosa *lung infection, when compared with Tob− group (Table 1). The 46 subjects (21.2 ± 9.0 (9–46) years) treated for* Pseudomonas aeruginosa *infections received a mean number of 6.3 ± 10.1 (1–57) intravenous antibiotic courses (corresponding to 44105 ± 80836 mg (520–480800 mg) or 487 ± 530 mg/kg (10–2890 mg/kg)) that included tobramycin given once daily. The targeted peak tobramycin level was 20–25 *μ*g/mL; the targeted trough tobramycin level was <2 *μ*g/mL [17]. The mean length of each antibiotic course was 13.5 ± 2.0 (3–21) days. The remaining 10 subjects with a mean age of 13.4 ± 5.3 (5–21) years were not treated with intravenous tobramycin (=Tob− group). Additionally, all but one subject of the Tob+ group and one subject of the Tob− group inhaled tobramycin for 1111 ± 1077 (1–4735) days. All subjects were well nourished. Clinical data are summarized in Table 1.

**Figure 1:**
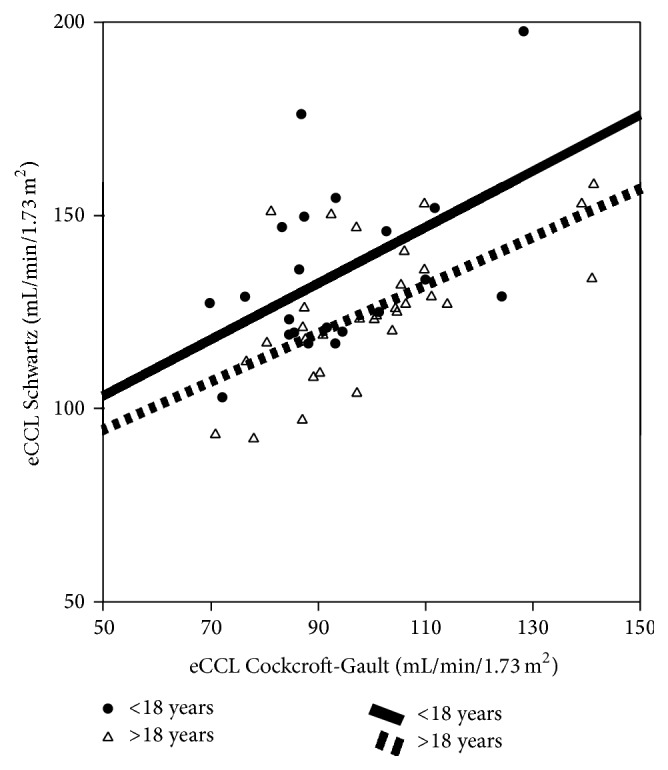
Correlation of estimated creatinine clearance (eCCL) calculated by the formula of Cockcroft-Gault versus formula by Schwartz (● = CF patients < 18 years, △ = CF patients > 18 years).

Serum creatinine was within the normal range for all subjects (in mg/dL: 0.9 ± 0.59 (0.2–1.1)). The mean eCCL values of all subjects calculated with the formula of Schwartz (119 ± 19 (92–236) mL/min/1.73 m^2^) was normal but mildly reduced after calculation with the Cockcroft-Gault formula (89 ± 21 (70–174) mL/min/1.73 m^2^). The eCCL of each individual subject calculated with the formula of Schwartz was normal. Employing the Cockcroft-Gault equation 22/56 (39%) had mildly decreased eCCL 69–90 mL/min/1.73 m^2^ (KDIGO G2) in the entire group. Only 17/46 (37%) of the subjects who received aminoglycosides had a mildly reduced eCCL, compared with 5/10 (50%) of the subjects who received no antibiotic treatment. Consistency of Cockcroft-Gault and Schwartz formula to estimate the creatinine clearance was poor (Pearson's *r* = 0.49, *p* < 0.001, Figure 1) with discrepancies in many subjects and a systematic overestimation of the eCCL by the Schwartz formula. Calculating the age dependent association of the Cockcroft-Gault eCCL and the Schwartz eCCL demonstrated a more pronounced overestimation in the <18 years age group (<18 years, *r* = 0.50, and *p* < 0.001; >18 years, *r* = 0.63, and *p* < 0.001, Figure 1). There was no correlation of eCCL and the number of tobramycin courses in group Tob+ (Cockcroft-Gault *r* = −0.17; Schwartz *r* = 0.03, Table 2).

**Figure 2:**
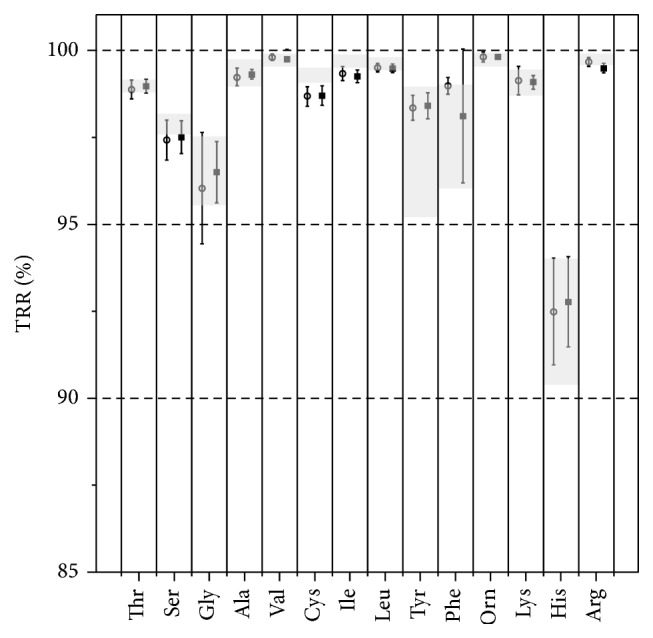
Renal tubular reabsorption of free amino acids (TRR%) compared between group Tob− (o = CF patients that never received intravenous tobramycin) and group Tob+ (■ = CF patients that received various courses of intravenous tobramycin). Gray shadowed areas represent the normal range.

Calculating the amino acid tubular reabsorption revealed no differences between subjects in the Tob+ and Tob− group using the Mann–Whitney *U* test (Figure 2). Employing Pearson's correlation we found a significant association between the amount of once-daily intravenous tobramycin courses and the tubular reabsorption of ornithine (*r* = −0.32, *p* = 0.02) but not with any other amino acid (Table 2). Further correlation to the absolute cumulative aminoglycoside dose confirmed an association with the tubular reabsorption of ornithine (*r* = 0.41, *p* < 0.01) but not with any other amino acid reabsorption. However, this association disappeared after correction for the weight dependent does (tobramycin in mg/kg: *r* = 0.05, *p* = 0.78). Inhalation of tobramycin was positively correlated to renal function estimated with the Cockcroft-Gault eCCL (*r* = 0.36, *p* < 0.01) and again an exclusive association with the tubular reabsorption of ornithine (*r* = 0.3, *p* = 0.03).

Univariate analysis of aminoglycoside tubular reabsorption adjusted for age revealed an age dependent association of leucine (*p* < 0.01) and isoleucine (*p* < 0.01) tubular reabsorption and repeated tobramycin courses. However, single Pearson's correlation did not reveal an association of age and tubular reabsorption of any amino acid, nor in the Tob+ or in the Tob− group.

## 4. Discussion

In the present study, we show that renal tubular function in patients with CF remained within normal ranges after repeated intravenous courses of tobramycin in the once-daily dosing scheme. ECCL was mildly reduced to KDIGO G2 in some patients employing the Cockcroft-Gault formula without any association with the frequency of intravenous tobramycin courses but was normal using the Schwartz formula. These results indicate that even frequent tobramycin treatments do not necessarily result in persistent kidney injury in patients with CF.

Data dealing with the impact of repeated aminoglycoside courses on GFR in patients with CF are controversial. Data from the Liverpool group [7] demonstrated that 31% of an adult CF cohort who received various numbers of aminoglycoside courses had a GFR <80 mL/min/1.73 m^2^. Impairment of renal function was significantly correlated to the cumulative aminoglycoside courses, but correlation coefficient was small (*r* = −0.32). A significant part of this cohort received 60 or more aminoglycoside courses for the treatment of a virulent multidrug resistant strain of* Pseudomonas aeruginosa* (named the Liverpool epidemic strain) which also resulted in a high frequency of acute renal failure in this cohort [7, 18]. In a Danish cohort [8] 40% of patients with an average of 20 aminoglycoside courses were reported to have an abnormal GFR, but no correlation (*r* = 0.11) between GFR and the cumulative tobramycin dose was observed. A more recent study on aminoglycoside pharmacokinetics observed no association of multiple aminoglycoside courses on renal function or aminoglycoside clearance [19]. Aminoglycoside antibiotics operate via a concentration dependent killing and a postantibiotic effect. Hence, once-daily dosing regimens were introduced that are considered less nephrotoxic [20]. This is the first study analysing renal function of CF patients exclusively treated with the once-daily regimen. We did not observe persistent glomerular or tubular dysfunction associated with multiple aminoglycoside courses. Renal histopathology studies reveal previously undiagnosed and variable renal pathologies associated with CF [5, 6]. Significant renal pathology other than nephrolithiasis or tubulointerstitial disease occurred in 2.5% of a large cohort of 510 adult patients with CF [6].

In accordance with these data we observed a mild—not clinically relevant—reduction of eCCL employing the Cockcroft-Gault formula with a frequency of 39% of our cohort. This is in the same range as previously reported [19] and disappeared using the Schwartz formula. The Cockcroft-Gault formula remains the favoured formula to estimate GFR in adult patients with normal serum creatinine but might be worse than other formulas like the modification of diet in renal disease formula in subjects with chronic kidney disease [21]. In our study correlation of the Cockcroft-Gault and Schwartz formula was poor (*r* = 0.49), which highlights the dilemma that no formula for eCCL is sufficient to estimate renal function in a CF cohort consisting of children, adolescents, and adult patients. In this transition cohort, calculation of renal function should not be done in an outpatient setting but based on a 24-hour urine collection.

Analysis of an association of renal function found a positive correlation of renal function estimated by the Cockcroft-Gault formula and dose of inhaled tobramycin which would indicate a positive effect of tobramycin inhalation on renal function in CF patients. As tobramycin might be resorbed in relevant concentrations [22], the expected effect would be negative. However, up to date chronic renal dysfunction after repeated courses of inhaled tobramycin was not observed [23].

Tubulointerstitial renal disease with lysosomal proliferation and tubular atrophy revealed in autopsies of patients with CF has been proposed to be caused by repeated aminoglycoside therapies [5]. It is widely accepted that proximal tubular dysfunction (Fanconi-like syndrome) usually is reversible after the aminoglycoside therapy is stopped [24]. Therefore we investigated renal tubular function with amino acid reabsorption in the interval between repeated tobramycin courses. We found an isolated association of reduced ornithine reabsorption and aminoglycoside courses and with leucine and isoleucine after age adjustment. Those are hardly true pathophysiologic phenomena as ornithine is reabsorbed in conjunction with arginine and lysine and leucine/isoleucine with valine [25], whose reabsorption was not associated with the frequency of tobramycin courses.

There are several reasons why patients with CF appear to be relatively resistant to aminoglycoside-induced nephrotoxicity. In particular children with CF have a higher volume of distribution for aminoglycoside antibiotics necessitating a higher dose compared to healthy children [26, 27]. The aminoglycoside courses applied in our study consisted exclusively of tobramycin, which is less nephrotoxic than gentamicin [28] and was applied in the once-daily dosing regimen that has reduced nephrotoxicity [20]. Due to higher patient weight and age, serum creatinine levels were higher in the patients who received tobramycin in this study.

## 5. Conclusion

In our study we demonstrate preserved glomerular and tubular function in patients with advanced CF who received repeated antibiotic treatment with tobramycin dosed once daily.

## Figures and Tables

**Table 1:** Patient characteristics.

Patient characteristics	Untreated (Tob−) group (mean ± SD)	Tobramycin-treated (Tob+) group (mean ± SD)	*p* value
Age (years)	13.4 ± 5.3 (5–21)	21.2 ± 9.0 (9–46)	0.01
FEV1 (% predicted)	87.4 ± 22.1 (59–131)	62.1 ± 22.1 (27–107)	0.002
BMI (kg/m^2^)	17.1 ± 1.8 (14.5–20.5)	18.9 ± 2.4 (14.5–24.1)	0.036
SDS-BMI	−0.8 ± 1.1 (−2.9–0.9)	−1.1 ± 0.8 (−2.8–0.8)	ns
Liver disease	1/10 (10%)	4/46 (9%)	ns
CFRDM	0/10 (0%)	5/46 (11%)	ns
Serum creatinine (mg/dL)	0.71 ± 0.17	0.81 ± 0.17	ns
eCCl Schwartz (mL/min/1.73 m^2^)	133 ± 27 (109–198)	130 ± 23 (92–236)	ns
GFR 69–90 mL/min/1.73 m^2^	0/10 (0%)	0/46 (0%)	ns
eCCl Cockcroft-Gault (mL/min/1.73 m^2^)	93 ± 18 (70–128)	99 ± 19 (71–174)	ns
GFR 69–90 mL/min/1.73 m^2^	5/10 (50%)	17/46 (37%)	ns
*P. aeruginosa*+	0/10 (0%)	46/46 (100%)	
iv tobramycin courses	0	6.3 ± 10.1 (1–57)	

Results are presented as mean ± SD (range). Untreated (Tob−) group: CF patients that never received intravenous AG antibiotics, Tobramycin-treated (Tob+) group: CF patients that received various courses of intravenous tobramycin antibiotics.

**Table 2:** Correlation of renal function, amino acid tubular reabsorption, and intravenous tobramycin courses in Tob+ group.

Amino acid	*r*	*p*
Serum creatinine (mg/dL)	0.15	0.27
eCCl Schwartz (mL/min/1.73 m^2^)	−0.07	0.63
eCCl Cockcroft-Gault (mL/min/1.73 m^2^)	−0.19	0.16
Threonine	−0.06	0.68
Serine	−0.03	0.83
Glycine	−0.02	0.86
Alanine	−0.06	0.68
Valine	−0.13	0.33
Cysteine	−0.04	0.79
Isoleucine	−0.18	0.20
Leucine	−0.12	0.39
Tyrosine	−0.05	0.70
Phenylalanine	0.01	0.97
Ornithine	−0.32	0.02
Lysine	−0.02	0.91
Histidine	−0.09	0.51
Arginine	−0.04	0.79

Correlation of renal function, amino acid tubular reabsorption, and intravenous tobramycin courses in Tob+ group. Results are presented as Pearson's correlation coefficient *r*.

## Abbreviations

AG: Aminoglycoside
CF: Cystic fibrosis
eCCL: Estimated creatinine clearance
GFR: Glomerular filtration rate.

## Additional Points

*Limitations of the Study*. The Tob+ and Tob− groups differ in regard of age, lung function, and BMI, which is the result of the more advanced stage of CF lung disease necessitating aminoglycoside therapies for* Pseudomonas aeruginosa* lung infection. Another limitation is the missing measured creatinine clearance with 24 hrs urine collection and the determination of serum cystatin C. We are unable to address the question of transient nephrotoxicity under or shortly after a course of aminoglycoside antibiotic in CF patients. Furthermore we solely present biochemical data but no histopathological work-up of a possible aminoglycoside-induced kidney injury.

## References

[1] Emerson J., Rosenfeld M., McNamara S., Ramsey B., Gibson R. L. (2002). Pseudomonas aeruginosa and other predictors of mortality and morbidity in young children with cystic fibrosis. *Pediatric Pulmonology*.

[2] Corris P. A. (2008). Lung transplantation for cystic fibrosis. *Current Opinion in Organ Transplantation*.

[3] Nazareth D., Walshaw M. (2013). A review of renal disease in cystic fibrosis. *Journal of Cystic Fibrosis*.

[4] Crawford I., Maloney P. C., Zeitlin P. L. (1991). Immunocytochemical localization of the cystic fibrosis gene product CFTR. *Proceedings of the National Academy of Sciences of the United States of America*.

[5] Abramowsky C. R., Swinehart G. L. (1982). The nephropathy of cystic fibrosis: a human model of chronic nephrotoxicity. *Human Pathology*.

[6] Yahiaoui Y., Jablonski M., Hubert D. (2009). Renal involvement in cystic fibrosis: diseases spectrum and clinical relevance. *Clinical Journal of the American Society of Nephrology*.

[7] Al-Aloul M., Miller H., Alapati S., Stockton P. A., Ledson M. J., Walshaw M. J. (2005). Renal impairment in cystic fibrosis patients due to repeated intravenous aminoglycoside use. *Pediatric Pulmonology*.

[8] Pedersen S. S., Jensen T., Osterhammel D., Osterhammel P. (1987). Cumulative and acute toxicity of repeated high-dose tobramycin treatment in cystic fibrosis. *Antimicrobial Agents and Chemotherapy*.

[9] Smyth A., Tan K. H.-V., Hyman-Taylor P. (2005). Once versus three-times daily regimens of tobramycin treatment for pulmonary exacerbations of cystic fibrosis—the TOPIC study: a randomised controlled trial. *The Lancet*.

[10] Glass S., Plant N. D., Spencer D. A. (2005). The effects of intravenous tobramycin on renal tubular function in children with cystic fibrosis. *Journal of Cystic Fibrosis*.

[11] Ghiculescu R. A., Kubler P. A. (2006). Aminoglycoside-associated Fanconi syndrome. *American Journal of Kidney Diseases*.

[12] Hanna M. H., Segar J. L., Teesch L. M., Kasper D. C., Schaefer F. S., Brophy P. D. (2013). Urinary metabolomic markers of aminoglycoside nephrotoxicity in newborn rats. *Pediatric Research*.

[13] Macpherson N. A., Moscarello M. A., Goldberg D. M. (1991). Aminoaciduria is an earlier index of renal tubular damage than conventional renal disease markers in the gentamicin-rat model of acute renal failure. *Clinical and Investigative Medicine*.

[14] Schwartz G. J., Muñoz A., Schneider M. F. (2009). New equations to estimate GFR in children with CKD. *Journal of the American Society of Nephrology*.

[15] Cockcroft D. W., Gault M. H. (1976). Prediction of creatinine clearance from serum creatinine. *Nephron*.

[16] (KDIGO) CKD Work Group (2013). KDIGO clinical practice guideline for the evaluation and management of chronic kidney disease. *Kidney International Supplements*.

[17] Vandenbussche H. L., Homnick D. N. (2012). Evaluation of serum concentrations achieved with an empiric once-daily tobramycin dosage regimen in children and adults with cystic fibrosis. *The Journal of Pediatric Pharmacology and Therapeutics*.

[18] Al-Aloul M., Miller H., Stockton P., Ledson M. J., Walshaw M. J. (2005). Acute renal failure in CF patients chronically infected by the Liverpool epidemic *Pseudomonas aeruginosa* strain (LES). *Journal of Cystic Fibrosis*.

[19] Alghanem S., Paterson I., Touw D. J., Thomson A. H. (2013). Influence of multiple courses of therapy on aminoglycoside clearance in adult patients with cystic fibrosis. *Journal of Antimicrobial Chemotherapy*.

[20] Smyth A. R., Bhatt J. (2012). Once-daily versus multiple-daily dosing with intravenous aminoglycosides for cystic fibrosis. *The Cochrane Database of Systematic Reviews*.

[21] Helou R. (2010). Should we continue to use the Cockcroft-Gault formula?. *Nephron—Clinical Practice*.

[22] Kahler D. A., Schowengerdt K. O., Fricker F. J., Mansfield M., Visner G. A., Faro A. (2003). Toxic serum trough concentrations after administration of nebulized tobramycin. *Pharmacotherapy*.

[23] Florescu M. C., Lyden E., Murphy P. J., Florescu D. F., Fillaus J. (2012). Long-term effect of chronic intravenous and inhaled nephrotoxic antibiotic treatment on the renal function of patients with cystic fibrosis. *Hemodialysis International*.

[24] Mingeot-Leclercq M.-P., Tulkens P. M. (1999). Aminoglycosides: nephrotoxicity. *Antimicrobial Agents and Chemotherapy*.

[25] Fotiadis D., Kanai Y., Palacín M. (2013). The SLC3 and SLC7 families of amino acid transporters. *Molecular Aspects of Medicine*.

[26] Levy J., Smith A. L., Koup J. R., Williams-Warren J., Ramsey B. (1984). Disposition of tobramycin in patients with cystic fibrosis: a prospective controlled study. *The Journal of Pediatrics*.

[27] Touw D. J., Vinks A. A. T. M. M., Neef C. (1997). Pharmacokinetic modelling of intravenous tobramycin in adolescent and adult patients with cystic fibrosis using the nonparametric expectation maximization (NPEM) algorithm. *Pharmacy World and Science*.

[28] Bertenshaw C., Watson A. R., Lewis S., Smyth A. (2007). Survey of acute renal failure in patients with cystic fibrosis in the UK. *Thorax*.

